# Computed Tomography-Guided Localization and Extended Segmentectomy for Non-Small Cell Lung Cancer

**DOI:** 10.3390/diagnostics12092043

**Published:** 2022-08-24

**Authors:** Wen-Yao Lee, Pei-Hsing Chen, Ke-Cheng Chen, Hsao-Hsun Hsu, Jin-Shing Chen

**Affiliations:** 1 Division of Thoracic Surgery, Department of Surgery, National Taiwan University Hospital, No. 7, Zhongshan S. Rd., Zhongzheng Dist., Taipei 100, Taiwan; 2 Department of Surgery, National Taiwan University College of Medicine, Taipei 100, Taiwan; 3 Division of Thoracic Surgery, Department of Surgery, National Taiwan University Hospital Yun-Lin Branch, No. 579, Sec. 2, Yunlin Rd., Douliu 640, Taiwan; 4 Department of Surgery, National Taiwan University Cancer Center, National Taiwan University College of Medicine, Taipei 106, Taiwan

**Keywords:** Lung cancer, VATS, thoracoscopy, Segmentectomy, CT-guided percutaneous needle localization

## Abstract

Background: Lung cancer is one of the most devastating cancers. Low-dose computed tomography (LDCT) can detect lung cancer at an early stage of the disease when a minimally invasive surgical procedure using video-assisted thoracoscopic surgery is the best strategy. Herein, we discuss the treatment of deep lung tumors between segments or lesions located near the margin of a segment. Patients and Methods: This was a retrospective study conducted from January 2013 to January 2020 using the National Taiwan University Hospital data bank. We included early-stage non-small cell lung cancer (NSCLC) patients who underwent lung surgery and screened out those who received CT-guided localization for extended segmentectomy. Outcome measurements were safety margin, complication rate, and postoperative course. Results: During the study period, 68 patients with early-stage NSCLC received CT-guided localization followed by extended segmentectomy. The mean surgery time was 92.1 ± 30.3 min, and the mean blood loss was 32.8 mL. Mean drainage time was 2.3 ± 1 days, and the total hospital stay was 4.9 ± 1.1 days. Pathological reports showed tumor-free resection margins >2 cm. Sixty-one patients had adenocarcinoma at stage IA and two patients at stage IB. One patient had squamous cell carcinoma at stage IA. Conclusion: CT-guided localization followed by extended segmentectomy allows lung volume preservation with clean safety margins and good clinical outcomes.

## 1. Introduction

Lung cancer has become a devastating cancer worldwide. Low-dose computed tomography (LDCT) is a promising screening test for its early diagnosis. The NELSON study confirmed the efficacy of LDCT screening for lung cancer while showing its value in decreasing mortality [1]. LDCT screening allowed the detection of many patients with early lung cancer. Since then, appropriate surgical resection for early non-small cell lung cancer (NSCLC) based on minimally invasive surgery with video-assisted thoracoscopic surgery (VATS) has become increasingly important. However, the precise identification of small nodules under the VATS remains challenging. With precise tumor localization, lesion resection preserving a higher lung volume while maintaining satisfactory oncological outcomes is possible.

Computed tomography (CT)-guided localization has been widely used for the resection of small pulmonary nodules [2,3]. This method resolves the problem of finding small nodules under VATS during surgery. Once the nodule is found, resecting it with safety margins and preserving lung volume are important concerns.

**Figure 1:**
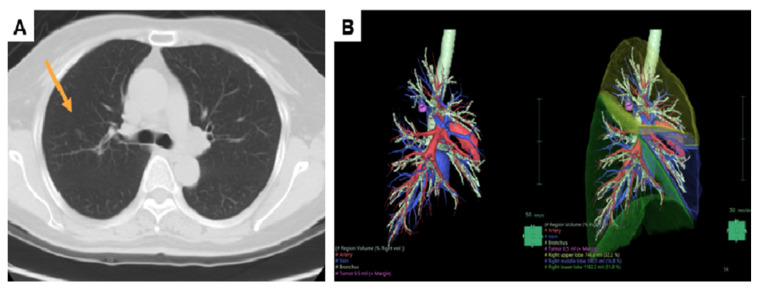
Characteristics of the lesion. (**A**) A deeply located lesion (arrow) over right upper lobe S2–S3 junction (**B**) 3D reconstruction of the lesion.

**Figure 2:**
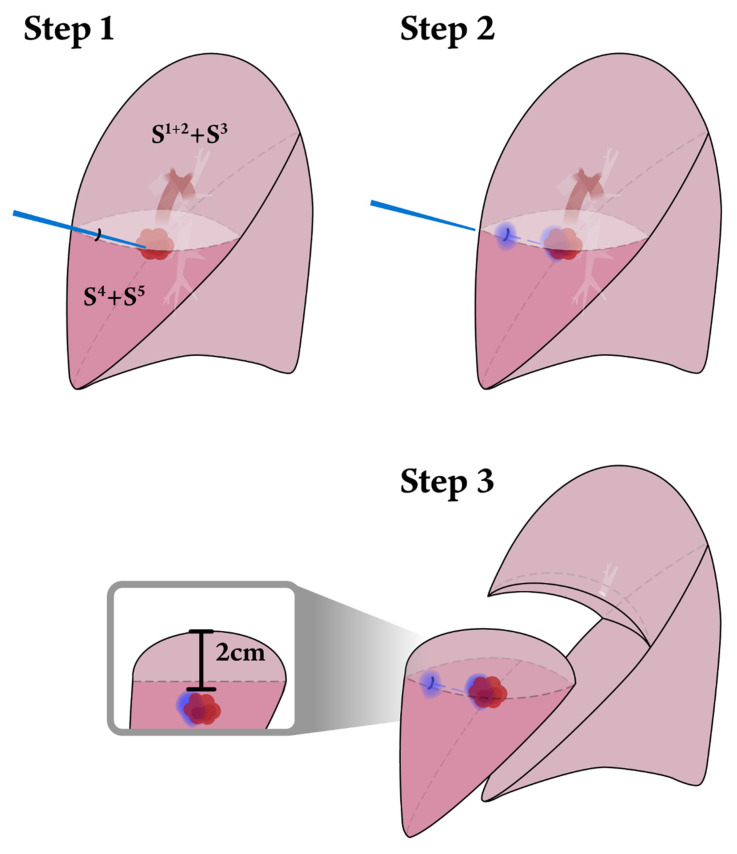
Operative procedures. **Step 1 and 2**: The blue line indicates the CT-guided dye localization located above the lesion. **Step 3**: With the help of the dye, segmentectomy can be performed with an extra margin for lesions over the intersegmental plane.

Sublobar resection is an appropriate procedure for ground glass opacity-dominant NSCLC at clinical stage IA [4]. Furthermore, no differences in short-term and long-term survival outcomes between the sublobar resection and lobectomy have been described [5,6,7,8,9]. According to the European Society of Thoracic Surgeons database, segmentectomy is preferably offered to compromised patients with limited respiratory function, higher American Society of Anesthesiologists (ASA) score, and relevant comorbidities [10]. This procedure showed a lower complication rate and similar long-term outcomes compared to lobectomy in stage I NSCLC [9]. As for VATS wedge resection, it might sacrifice more lung volume when resecting deeper lesions under uniportal VATS, while standard VATS single segmentectomy might not gain a suitable margin if the nodules are close to another segment (such as a lesion near the right upper lobe S2–S3 junction) (Figure 1). In such cases, segmentectomy with more than one segment is a usual operative method. However, another concern may be lung volume wasting. Extended segmentectomy is an alternative to increase safety margins during pulmonary nodule resection [11]. We defined extended segmentectomy as segmentectomy with a wedge resection of a neighbor segment (Figure 2).

CT-guided localization marks the small pulmonary nodule lesion, and extended segmentectomy allows suitable safety margins during VATS [5,11]. Therefore, combining these two methods for deeper lesions near the adjacent segment would be ideal. However, to date, there are no reports on CT-guided localization followed by extended segmentectomy. Herein, we describe the feasible surgical intervention of CT-guided localization combining extended segmentectomy to help us find and resect small pulmonary lesions with adequate margins in a retrospective study.

## 2. Materials and Methods

### 2.1. Study Population

**Figure 3:**
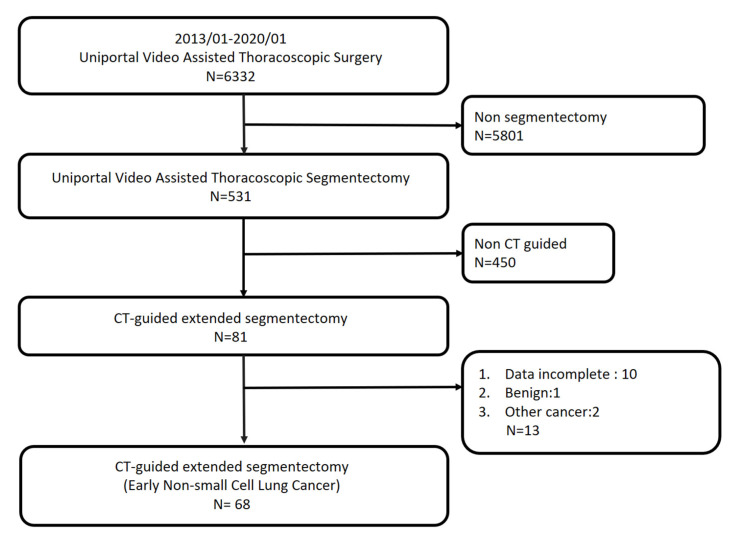
Algorithm for patient selection.

This was a retrospective study from January 2013 to January 2020 using data from the National Taiwan University Hospital database. We included patients who underwent uniportal thoracoscopic lung surgery and selected those who received CT-guided localization followed by segmentectomy. During the study period, 6362 patients received uniportal VATS surgery at the hospital. Among them, 534 patients underwent segmentectomy. Overall, 84 patients were scheduled to receive preoperative CT-guided localization for uniportal VATS segmentectomy. Ten patients were excluded had incomplete data, one patient had a benign tumor, three patients had operative methods that were changed to wedge resection, and two patients had metastatic cancer. Overall, 68 patients who received CT-guided localization followed by extended segmentectomy with a final pathology diagnosis of NSCLC were included (Figure 3). The institutional review board number is 202102046RINA.

### 2.2. Technique for Lung Nodule Marking

Before surgery, patients were sent to the CT room previously on the same day. There, the patient underwent an initial CT scan to determine the location of the pulmonary nodule. Then, the radiologist inserted a 22 Gauge Chiba needle into the nodule under CT guidance. Next, about 0.1–0.3 mL methylene blue dye was injected. For deep lesions, the radiologist performed dual die injections with about 0.2 mL over the lesion and 0.1 mL in the subpleural area. The dye and pinhole on the lung surface were useful for tumor orientation, while that at deep locations near the tumor helps quickly locate the tumor and ensure an adequate margin during lung lesion resection. A deeper dye was used to adjust our stapler when the dye was encountered during lung resection. After the procedure, we repeated a CT scan to check the dye distribution and discard iatrogenic pneumothorax or hemothorax. If no iatrogenic problem was found, the patient was sent back to the general ward to await surgery.

### 2.3. Technique for Uniportal VATS Segmentectomy

After CT-guided localization, the patient was sent to the operation room. The patient was placed in the lateral decubitus position after anesthesia induction. A single skin incision was performed at the anterior axillary line along the 5th intercostal space, and a wound protector was applied at the incision site. A 5 mm, 30-degree video telescope (Karl Storz, Tuttlingen, Germany and Olympus, Tokyo, Japan) was used during surgery. Curved-tip endovascular staplers (Johnson & Johnson Institute, Cincinnati, Ohio) were used to separate the segmental arteries and veins. An endo-stapler (Johnson & Johnson Institute, Cincinnati, Ohio and Medtronic, Minneapolis, MN, USA) was used to separate the bronchus and lung parenchyma. The intersegmental plane of the lung parenchyma was determined depending on the dye marking. Then, the extended segmentectomy (Figure 2) was performed [5,11].

### 2.4. Postoperative Care

The patients were able to resume oral intake within 4 h in the intubated group, or within 2 h in the non-intubated group postoperatively. Postoperative pain control was provided by regular oral nonsteroidal analgesics or acetaminophen. Patient-controlled analgesia with morphine (1 mg/mL) was administered intravenously, as required by the patients. A chest X-ray was performed in the first postoperative day. The pleural cavity drainage tube was removed in the absence of air leak and <200 mL drainage in a 24-h period. All postoperative complications were recorded in detail. A prolonged air leak was defined as air leakage time >3 days postoperatively.

## 3. Results

### 3.1. Patient Demographics

During the study period, 68 patients (16 men and 52 women) with early-stage NSCLC received CT-guided localization followed by uniportal VATS extended segmentectomy. The median age was 58.7 ± 10.8 years and the median Body Mass Index (BMI) was 23.3 ± 3.1(kg/m^2^). The median pulmonary function test showed forced expiratory volume in one second (FEV1) as 109 ± 16% and forced vital capacity (FVC) as 108.5 ±17.7%. More than ninety percent of the CT scan valued tumor sizes were <20 mm, with a mean depth of skin to the lesion (Depth 1) of 6.5 ± 1.6 cm, and chest wall to the lesion (Depth 2) of 3.0 ± 1.4 cm. There were 22 patients in ASA class I, 45 patients in ASA class II, 1 patient in ASA class III, and 0 patient in ASA class IV (Table 1).

### 3.2. Types of Segmentectomy

All patients in this study received CT-guided localization because of the difficulty to precisely localize the pulmonary nodule by CT scan before surgery. Forty-six patients received left side VATS extended segmentectomy. The most operative methods included S1 extends to S3 (n = 11) and S6 extends to S10 segmentectomy (n = 11). The others are presented in Table 2. The pulmonary nodules in two patients located between the S8 + 9 + 10 in the right lower lobe and S8 + 9 + 10 in the left lower lobe, received VATS common basal segmentectomy directly because the lesion was too deep (Table 2).

### 3.3. Perioperative and Postoperative Outcomes and Histology

In this study, 15 patients received non-intubated CT-guided localization uniportal VATS extended segmentectomy. The non-intubated procedure was based on the Anesthesiologist’s protocol from the National Taiwan University Hospital [12,13]. The mean operative time was 92.1 ± 30.3 min, with a mean blood loss of 29 ± 2 mL. Regarding the postoperative course, the mean drainage time was 2.3 ± 1 days, and the total hospital stay was 4.9 ± 1.1 days. Six patients had prolonged air leakage and one patient had postoperative pneumonia, cured under conservative treatment. All patients had disease-free survival (100%) during the follow-up period. As for pathology reports, the tumor-free resection margins were all >2 cm. These reports showed 61 patients with adenocarcinoma at clinical stage IA, 2 patients at IB, and 4 patients with adenocarcinoma in situ (stage 0). 1 patient had squamous cell carcinoma at clinical stage IA (Table 3).

## 4. Discussion

In the LDCT era, lung cancer is increasingly being identified by screening at early stages. However, the management of deeply located early-stage lung cancer remains challenging. Previously, lobectomy was required to resect deep-seated lung cancer. However, with our current novel method, by using CT-guided dual dye localization, a minor procedure including segmentectomy may be sufficient. The dual dye and pinhole on the lung surface can help surgeons decide the direction and depth while applying an endo-GIA stapler. Further, the deeply located dye near the tumor enhances precise tumor localization, as well as intersegmental margin identification during further lung stapling. The average distance between the lesion and the lung surface is 3 cm, suggesting that the lesion is close to the pulmonary hilum in our cases. The deeper marker near the tumor is used for the safety margin. In other words, when we cut the lung parenchyma and dissect the intersegmental plane, and if we see the blue color around the stapling line, thus we can realize that the margin may not be sufficient; but if there is no blue color around the stapling line, then we realize that the margin is adequate.

Few studies have discussed the methods employed to locate small lung tumors. Although other advanced tools for localization, such as hook wires or microcoils, were reported to be safe and efficient for VATS surgery [14,15]. Our method with CT-guided dye localization followed by uniportal VATS extended segmentectomy for small pulmonary nodules is also a feasible approach.

Technological advances, such as 3D reconstruction imaging interpretation and analysis, can facilitate complicated segmentectomy [16,17,18] by providing more information to clarify the segmental margin and design the surgical plane for intersegmental lesions. 

Recently, hybrid operating rooms have been widely used because of single-stage localization and image-guided VATS for the removal of small pulmonary nodules [1,19,20]. However, cost considerations may become the primary driving factor favoring the significantly shorter operating room use times with preoperative procedures [18]. The potential use of localization procedures utilizing electromagnetic navigation bronchoscopy, intraoperative ultrasound, and real-time on-table cone-beam CT scans during image-guided thoracoscopic surgery without lung puncture may offer superior localization of small pulmonary nodules to reduce the risks, while patients are spared the compounding wait times and follow-up appointments [20]. For cases with tumors located extremely deep and near the bronchus, dye localization may have a risk of complications, including bleeding or air emboli [21]. In such situations, in the hybrid operating room, virtually assisted lung mapping, which can perform localization through electromagnetic navigation bronchoscopy, may play a significant role [19,20,22,23,24,25].

To increase lung volume preservation and assure more precise lung surgery, localization is critical. More patients could receive surgery and improve their life quality with good clinical outcomes. The easier and standard operation of lobectomy was excessive for small lung nodules, and with our method, we could get appropriate safety margin and lung volume preservation. Despite the limitations for tumors near the main bronchus or vessels, lobectomy still has a role in certain clinical situations. In the future, we will be able to choose among various methods depending on tumor location and clinical situation to design the most feasible treatment plans for patients. Using our method, 15 patients needed only nonintubated anesthesia for the operation [12,13]. With adequate patient selection, CT-guided localization followed by non-intubated uniportal VATS segmentectomy could cause less damage to the patient during surgery and accelerate recovery.

This study was limited by its retrospective design and the small number of patients. Meanwhile, the lack of a control group and short-term follow-up makes it difficult to differentiate the specific benefits of further oncologic outcomes. Therefore, further studies with a larger number of patient samples and long-term follow-ups are needed to confirm or dismiss our results. Besides, the learning time for this technique is significant.

## 5. Conclusions

For deeply seeded lung tumors less than2 cm with a partially solid component located between the intersegmental planes, designing the surgical plane for precise lung surgery to preserve a high lung volume remains critical. In this study, we presented a feasible way of accomplishing this goal by using CT-guided dual dye localization followed by extended segmentectomy. This method may preserve a higher lung volume with adequate safety margins and good clinical outcomes in selected patients. Moreover, a better quality of life may be achieved in compromised patients with old age, limited pulmonary function, or cardiovascular disease.

## Figures and Tables

**Table 1:** Demographic and clinical features.

Variable	Result No. (%), or Mean (±SD)
Age, y	58.7 ± 10.8
Male	16 (23.5%)
Height (cm)	159.4 ± 6.9
Body weight (kg)	59.2 ± 8.6
BMI (kg/m^2^)	23.3 ± 3.1
Pulmonary function test, % of prediction	
Forced expiratory volume in 1 s	109.0 ± 16
Forced vital capacity	108.5 ± 17.7
CT nodule size (cm)	1.2 ± 0.5
<10 mm	25 (36.8%)
≥10 mm but <20 mm	39 (57.4%)
≥20 mm	4 (5.9%)
Depth 1 (cm) (Skin to lesion)	6.5 ± 1.6
Depth 2 (cm) (Chest wall to lesion)	3.0 ± 1.4
Comorbidity	
HTN	9 (13.2%)
DM	5 (7.4%)
Hyperlipidemia	5 (7.4%)
COPD and asthma	4 (5.9%)
HBV	8 (11.8%)
Previous lung cancer	4 (5.9%)
Other cancers	9 (13.2%)
ASA class	
I	22 (32.5%)
II	45 (66%)
III	1 (1.5%)
Ⅳ	0 (0%)

BMI, body mass index; Depth 1 and Depth 2, the distance was valued from CT scan; HTN, hypertension; DM, diabetes mellitus; COPD, chronic obstructive pulmonary disease, HBV, hepatitis B Virus, ASA, American Society of Anesthesiologists.

**Table 2:** Lesion distribution by segments.

Lobe	Tumor Location by CT Scan (Segment)	No. (%)	Operative Method	No. (%)
RUL (N = 10)				
	S1 + 2	1 (1.4%)	S2 extends to S1	1 (1.4%)
	S1 + 3	5 (7.4%)	S3 extends to S1	5 (7.4%)
	S2 + 3	4 (5.9%)	S2 extends to S3	1 (1.4%)
			S3 extends to S2	3 (4.4%)
RML (N = 1)				
	S4 + 5	1 (1.4%)	S5 extends to S4	1 (1.4%)
RLL (N = 11)				
	S6 + 8	3 (4.4%)	S6 extends to S8	3 (4.4%)
	S6 + 9 + 10	1 (1.4%)	S6 extends to S9 and 10	1 (1.4%)
	S6 + 10	5 (7.4%)	S6 extends to S10	5 (7.4%)
	S9 + 10	1 (1.4%)	S9 extends to S10	1 (1.4%)
	S8 + 9 + 10	1 (1.4%)	Common basal segment	1 (1.4%)
LUL (N = 24)				
	S1 + 2	2 (2.9%)	S2 extends to S1	2 (2.9%)
	S1 + 3	11 (16.2%)	S1 extends to S3	11 (16.1%)
	S2 + 3	3 (4.4%)	S2 extends to S3	2 (2.9%)
			S3 extends to S2	1 (1.4%)
	S3 + 4	5 (7.3%)	Proper segment extends to S4	2 (2.9%)
			Lingular segment extends to S3	3 (4.4%)
	S4 + 5	3 (4.4%)	S5 extends to S4	3 (4.4%)
LLL (N = 22)				
	S6 + 8	3 (4.4%)	S6 extends to S8	3 (4.4%)
	S6 + 9	2 (2.9%)	S6 extends to S9	2 (2.9%)
	S6 + 10	11 (16.2%)	S6 extends to S10	11 (16.2%)
	S8 + 9	5 (7.4%)	S8 extends to S9	5 (7.4%)
	S8 + 9 + 10	1 (1.4%)	Common basal segment	1 (1.4%)

RUL, right upper lobe; RML, right middle lobe; RLL, right lower lobe; LUL, Left upper lobe; LLL, Left lower lobe; S, Segment.

**Table 3:** Short-term postoperative outcomes and pathology.

Variable	Result No. (%), or Mean (±SD)
Non-intubated	15 (22.1%)
Operative time (min)	92.1 ± 30.3
Blood loss (mL)	29 ± 2
Drain duration (days)	2.3 ± 1
Hospital stays (days)	4.9 ± 1.1
Complication	
Prolonged air leakage	6 (8.8%)
Pneumonia	1 (1.4%)
Margin involvement	0
R0 resection	68 (100.0%)
Pathologic stage	
Adenocarcinoma	
Adenocarcinoma in situ (stage 0)	4 (5.9%)
IA	61 (89.7%)
IB	2 (2.9%)
Squamous cell carcinoma	
IA	1 (1.4%)
